# Electrospun nanofiber templated assembly of hybrid nanoparticles†

**DOI:** 10.1039/c8ra00665b

**Published:** 2018-03-05

**Authors:** Zhicheng Liu, Zhaodong Yan, Lu Bai

**Affiliations:** School of Materials Science and Engineering, North University of China Taiyuan 030051 China; School of Chemical Engineering and Technology, North University of China Taiyuan 030051 China bailu0919@gmail.com; Department of Mechanical Engineering, National University of Singapore Singapore 117574 Singapore

## Abstract

Assembling nanoparticles into or onto a three-dimensional template such as an electrospun nanofiber membrane has attracted considerable attention since this composite material has great potential in many applications. We report here that hybrid noble metal nanoparticles could be readily assembled both into and onto electrospun nanofibers using simple mixing and immersion steps. It is observed that small gold nanospheres were well distributed within the nanofiber, while other nanoparticles such as big gold nanospheres, gold nanorods and palladium nanocubes were uniformly decorated on the surface of the nanofibers. Moreover, the hybrid nanoparticle-assembled nanofiber membrane showed impressive SERS and catalytic performance based on the type of the assembled nanoparticles. It is believed that other nanomaterials could also be assembled with nanofiber membranes using this facile strategy.

## Abstract 2

Hybrid noble metal nanoparticles could be readily assembled both into and onto electrospun nanofibers.

## Graphical abstract

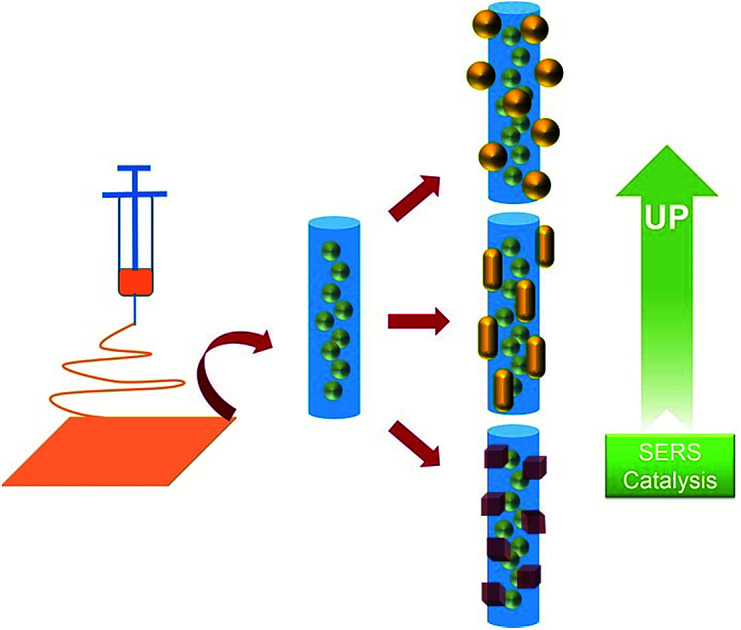

## Introduction

In recent years, in the fields of chemistry and materials, there has been growing research interest regarding noble metal nanoparticles (NMNPs) due to their unique optical, electrical and catalytic properties.^1,2^ Because of their high surface energy, they tend to aggregate in solution or during confinement, which greatly restrains their potential in many applications. Assembling the NMNPs into or onto a proper template is one of the most effective methods to solve the problem. Up to now, the NMNPs have been assembled into or onto one-, two- and three-dimensional templates.^3,4^ In particular, three-dimensional (3D) templated assembly of NMNPs has shown potential in many fields such as optics, surface enhanced Raman scattering (SERS), electrocatalysis and biomedicine.^5^

Electrospinning is a remarkably efficient and straightforward approach to prepare large-scale 3D polymer nanofiber membrane.^6,7^ The freestanding electrospun nanofiber membranes with the advantages of high porosity and high surface area to volume ratio have been recognized as perfect substrates to assembly NMNPs.^8,9^ Generally, the NMNPs could be assembled either into or onto the electrospun nanofibers. For example, both silver NPs (Ag NPs) and gold nanorods (Au NRs) could be readily assembled into electrospun nanofibers by mixing the NP solution with the polymer solution.^10–13^ On one hand, the NPs are protected by the surrounding polymers from aggregation, and on the other hand, the chemicals or analytes should diffuse through the polymer barrier to access the active NPs. In other cases, NMNPs could be directly assembled onto the surface of electrospun nanofibers through various kinds of interactions such as electrostatic interaction and hydrogen bonding.^14–18^ For instance, by taking advantage of the hydrogen bonding interaction between the amide groups on the nanofibers and the carboxylic acid groups on the NPs, Au NPs were successfully decorated on the surface of the polyamide nanofiber.^14^ It is worth mentioning that there are few reports showing the assembly of two or more kinds of NMNPs into or onto electrospun nanofibers, although there might be new or improved properties because of the increased NP loading and the possible synergistic effect. Recently, Zhang and Yu presented that Au NRs could be assembled on the Ag nanowires (NWs) to form an Au NR–Ag NW nanocomposite.^19^ Then the nanocomposites were electrospun into nanofiber membranes with different optical properties and enhanced SERS activity. Given that little effort has been made in this subject, it remains a challenge to design and fabricate 3D electrospun nanofiber membranes assembled with different kinds of NMNPs.

In this work, composite nanofiber membranes with diverse metal NPs assembled both into and onto the nanofibers were facilely prepared. Firstly, small Au NPs were assembled inside the electrospun poly(acrylic acid)/poly(vinyl alcohol) (PAA/PVA) nanofibers by mixing the Au NP solution with the polymer solution. Then, driven by electrostatic interaction, both sphere-like and anisotropic positively-charged NMNPs were immobilized outside the nanofiber through a simple immersion step. Moreover, due to the high loading of NMNPs, these 3D NPs-assembled nanofiber membranes showed excellent SERS and catalytic property.

## Experimental

### Materials

PAA (*M*_w_ = 240 000) was obtained from J&K Scientific (Beijing, China). PVA (PVA-2088) was purchased from Chenqi Chemical Technology Co., Ltd (Shanghai, China). Sodium borohydride (NaBH_4_) was purchased from Macklin Biochemical Co., Ltd (Shanghai, China). Other chemicals such as 4-aminothiophenol (4-ATP) were from Sinopharm Chemical Reagent Co., Ltd (Shanghai, China). All chemicals were used as received without further purification.

### Synthesis of Au nanoparticles (Au NPs)

Au NPs with different sizes were synthesized using the classical seeded growth method.^20^ The seed solution was prepared by adding 0.6 mL of ice-cold and freshly prepared 0.1 M NaBH_4_ into 20 mL mixed solution of trisodium citrate (2.5 × 10^−4^ M) and chloroauric acid (HAuCl_4_, 2.5 × 10^−4^ M). The color turned pink, suggesting the formation of Au seeds. To prepare the growth solution, HAuCl_4_ (2.5 × 10^−4^ M) and cetyltrimethylammonium bromide (CTAB, 0.08 M) were mixed in 100 mL aqueous solution, and 9 mL of the growth solution and 0.05 mL of 0.1 M ascorbic acid solution were mixed. Subsequently, 1 mL of the seed solution was added under vigorously stirring. The mixed solution was used as seed solution for the next growth process in 30 min. The small and big Au NPs (denoted as Au sNPs and Au bNPs) were finally obtained after one and two more similar growth processes, respectively.

### Synthesis of Pd nanocubes (Pd NCs)

The CTAB-protected Pd NCs were synthesized as previously described.^21^ In brief, CTAB (0.1820 g) and sodium ascorbate (0.0099 g) were mixed in 15 mL of deionized water, and the solution was stirred at 50 °C. Palladium nitrate dehydrate (0.0108 g) was dissolved in 5 mL of deionized water, and then those solutions were rapidly mixed together. The synthesis was completed in about 30 min.

### Synthesis of Au nanorods (Au NRs)

Au NRs were prepared according to the classic seed-mediated growth method.^22^ Firstly, HAuCl_4_ (2.5 × 10^−4^ M) and CTAB (0.1 M) were mixed in 10 mL aqueous solution, and 0.6 mL of ice-cold and freshly prepared 0.1 M aqueous NaBH_4_ solution were added. The resulted solution could be used as seed solution for the growth of Au NRs. Secondly, the growth solution was prepared by adding 2 mL 0.01 M HAuCl_4_, 500 μL 0.01 M AgNO_3_ and 300 μL 0.1 M ascorbic acid solution into 47.5 mL 0.1 M CTAB aqueous solution. Finally, 60 μL seed solution was added to the growth solution by hand shaking, and then the solution was left undisturbed overnight to grow Au NRs.

### Preparation of NMNPs assembled PAA/PVA electrospun nanofibers

The Au sNPs were assembled inside the PAA/PVA electrospun nanofiber through simple solution blending. Briefly, the Au sNPs solution was concentrated 100-fold by centrifugation, and the solution was used to prepare a 10 wt% PAA/PVA solution. Then the hybrid solution was loaded into a syringe with a 22-gauge blunt tip needle and electrospun into nanofibers. The applied voltage was 15 kV while the flow rate was 300 μL h^−1^. The collection distance was 25 cm. The electrospinning process was finished after 1.5 h, and the resulted nanofibrous membrane was crosslinked by heat treatment at 145 °C for 30 min to produce water-stable nanofiber membrane. The membranes were immersed in the Au bNPs solution, the Au NRs solution and the Pd NCs solution for 12 h to assemble NMNPs, respectively. The membranes are also denoted as Au sNPs–Au bNPs, Au sNPs–Au NRs and Au sNPs–Pd NCs composite nanofiber membranes, respectively. Finally, these membranes were washed with water to remove the loosely bound NMNPs and left to dry in air.

### Catalytic reduction of 4-nitrophenol

The catalytic activity of the Au sNPs–Pd NCs composite nanofibers toward 4-nitrophenol reduction was tested. A solution mixture containing 4-nitrophenol (5 mL, 1 mM) and NaBH_4_ aqueous solution (5 mL, 0.1 M) was prepared in a 25 mL beaker. Then 35 mg of the nanofibrous membrane was immersed into the solution at room temperature. The reaction rate of the 4-nitrophenol reduction was evaluated using UV-vis spectroscopy.

### Characterization

The NPs and the NPs-assembled nanofibers were observed using a transmission electron microscopy (TEM, JEOL-2100F) with an acceleration voltage of 200 kV. The morphologies of the NPs-assembled nanofibers were also observed by a scanning electron microscopy (SEM, Hitachi SU8010). The UV-vis absorption spectra were measured by a Unico UV-4800 UV-vis spectrometer. The X-ray diffraction (XRD) patterns were obtained from a DX-2700 X-ray diffractometer. The thermogravimetric (TG) experiments were performed on a TA Q 50 thermogravimetric analyzer. The metal content was measured using an inductively coupled plasma (Thermo Fisher Scientific, iCAP-6300) instrument. The SERS performance of the nanofibrous membranes was measured by a Renishaw InVia confocal Raman spectrometer using 4-aminophenol as probing molecule. All SERS spectra were recorded using 785 nm excitation, and the excitation power was 0.15 mW. The integral time was 10 s.

## Results and discussion

**Fig. 1:**
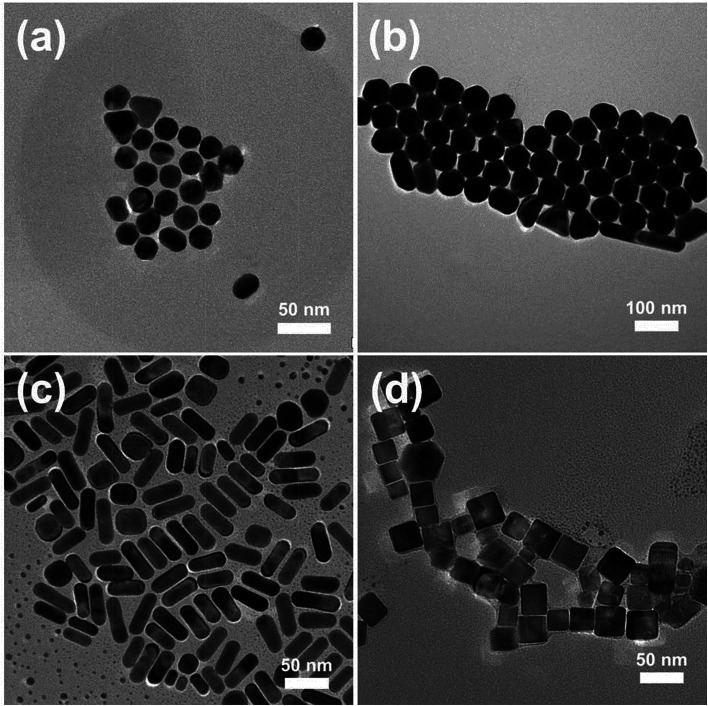
TEM images of (a) Au sNPs, (b) Au bNPs, (c) Au NRs and (d) Pd NCs.

The assembly of NPs both inside and outside the PAA/PVA nanofiber was achieved through a two-step assembly process. The first step is the mixing of the NP solution with the PAA/PVA solution to be electrospun, and the second step is the immersion of the NP assembled nanofibers into another NP solution. In order to assemble different kinds of NMNPs on 3D electrospun nanofiber membranes, Au NPs with different sizes, Au NRs as well as Pd NCs were synthesized. The typical TEM images of the CTAB-protected NPs and their UV-vis spectra are shown in Fig. 1 and S1,† respectively. The Au NPs (Fig. 1a and b) with diameters of about 23 nm (Au sNPs) and 62 nm (Au bNPs) were successfully obtained. The peaks of the UV-vis spectra located at 522 nm and 545 nm arise from the surface plasmon resonance of Au NPs.^23^ As shown in Fig. 1c, the as-synthesized Au NRs are about 46 nm in length and 17 nm in width (average aspect ratio was ∼2.7). The obvious absorption peaks at 520 nm and 730 nm (Fig. S1c†) are typical peaks of Au NRs due to the well-known transverse and longitudinal plasmon resonance.^23^ Besides Au NRs, another kind of classic anisotropic NPs, *i.e.* Pd NCs were also prepared, as displayed in Fig. 1d. The cubical NPs had an edge length of about 26 nm. It is worth noting that all the NMNPs were protected by positively-charged CTAB molecules. The capped CTAB bilayer could not only prevent the NPs from aggregation but also play an important role in assembling the NPs into and onto the electrospun nanofibers. The interaction between the positively-charged CTAB and the negatively-charged PAA could promote the dispersion of Au NPs in the PAA/PVA solution, which leads to the uniform NP assembly in the as-spun nanofibers. Moreover, the electrostatic interaction is the driving force for the following NP assembly on the PAA/PVA nanofibers.

In order to obtain relatively uniform nanofibers, the distance between the needle tip and the collector was adjusted. As shown in Fig. S3,† the decrease in needle-to-collector distance leads to an increase in the polydispersity of nanofibers diameters, which might be ascribed to the incomplete evaporation of the solvent and the insufficient bending time of the emitted nanofibers. When the distance was above 25 cm, the relatively uniform nanofibers which could be used to clearly demonstrate the NP assembly were acquired.

**Fig. 2:**
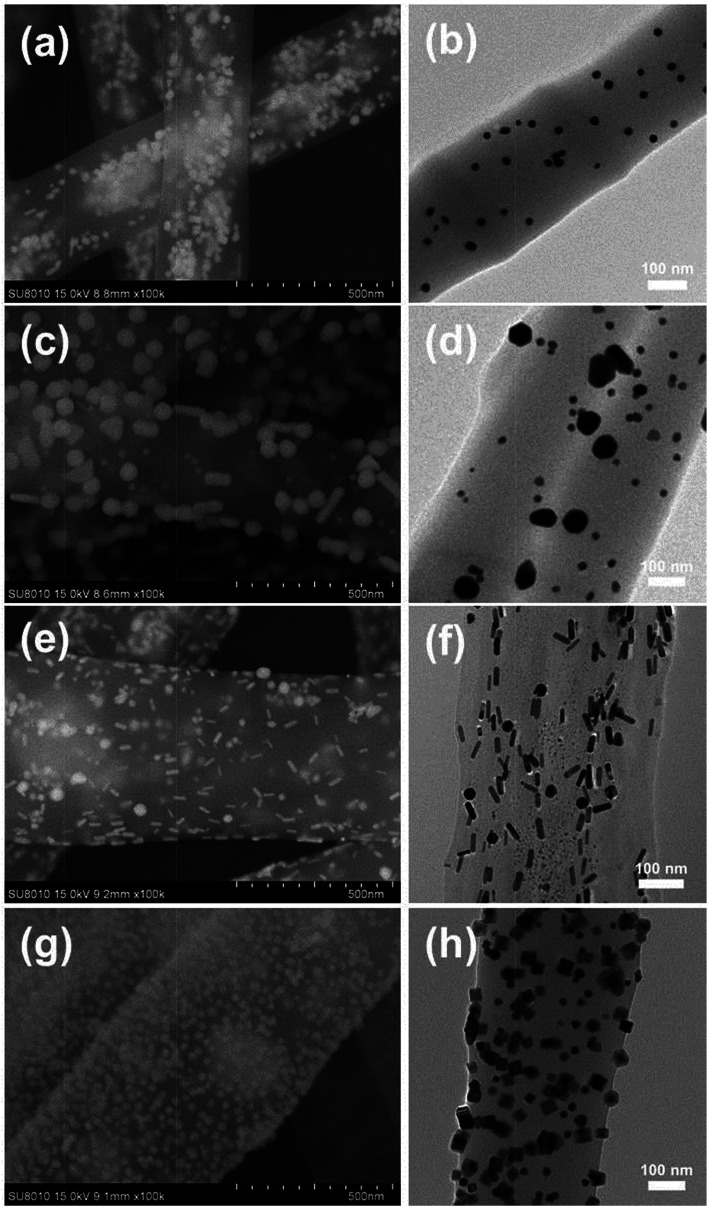
SEM and TEM images of the PAA/PVA electrospun nanofibers assembled with (a and b) Au sNPs, (c and d) Au sNPs–Au bNPs, (e and f) Au sNPs–Au NRs and (g and h) Au sNPs–Pd NCs.

Fig. 2 shows the SEM and TEM images of different NMNPs assembled PAA/PVA electrospun nanofibers. Obviously, all these four kinds of NPs were successfully assembled in or on the nanofibers in a relatively uniform fashion. Fig. 2a and b present the distribution of Au sNPs in the PAA/PVA nanofibers, and the Au sNPs-assembled nanofibers are about 400 nm in diameter. Since both PAA and PVA are water-soluble polymers, a thermal treatment was conducted to crosslink the electrospun nanofibers before the assembly of NMNPs onto the nanofibers.^15,24,25^ The esterification reaction (during the thermal treatment) between the carboxylic acid of PAA and the hydroxyl groups of PVA renders the PAA/PVA electrospun nanofibers insoluble in water. After immersing in different NMNPs solution, Au bNPs (Fig. 2c and d), Au NRs (Fig. 2e and f) and Pd NCs (Fig. 2g and h) were successfully assembled on the surface of the Au sNPs-assembled PAA/PVA nanofibers. The diameters of these nanofibers changed to about 500 nm due to the swelling effect of the immersion step. It is noteworthy that there were no significant changes of size and shape of the assembled Au sNPs after the thermal treatment. Hence, NMNPs with different sizes (Au sNPs–Au bNPs), different elemental compositions (Au sNPs–Pd NCs) and different shapes (Au sNPs–Au NRs and Au sNPs–Pd NCs) were facilely assembled both into and onto the PAA/PVA nanofibers. Large area 3D NPs-assembled nanofiber membranes were achieved, as shown in Fig. S2 and S4.† It is anticipated that other functional nanomaterials could also be assembled with the nanofiber membranes through proper design.

**Fig. 3:**
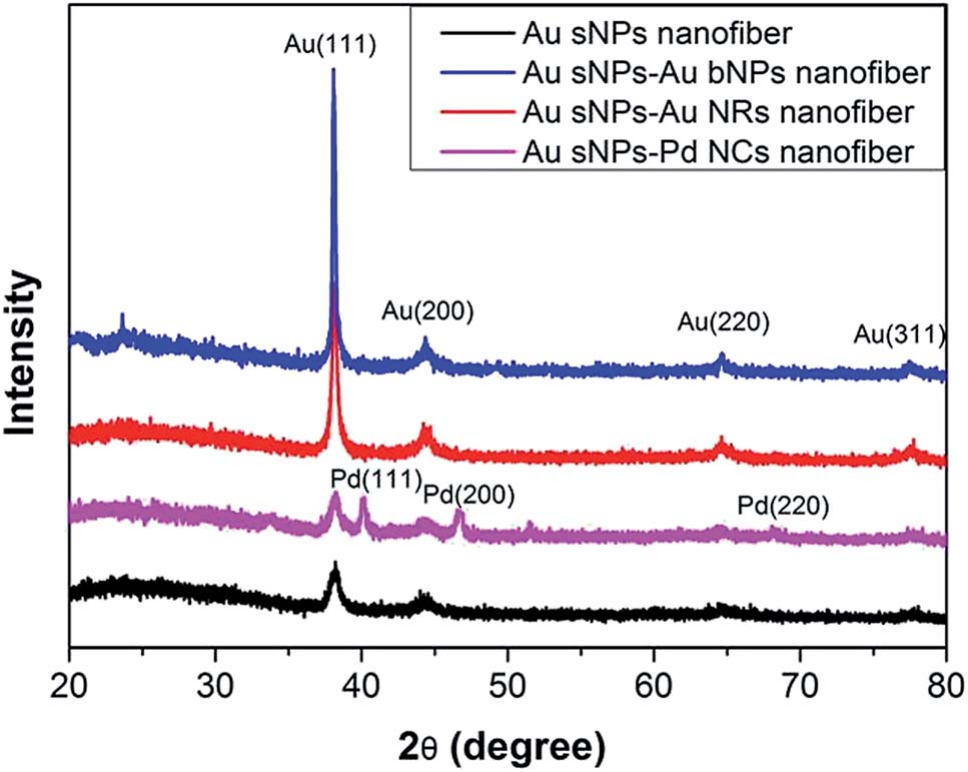
XRD patterns of the PAA/PVA nanofibers assembled with Au sNPs, Au sNPs–Au bNPs, Au sNPs–Au NRs and Au sNPs–Pd NCs, respectively.

**Fig. 4:**
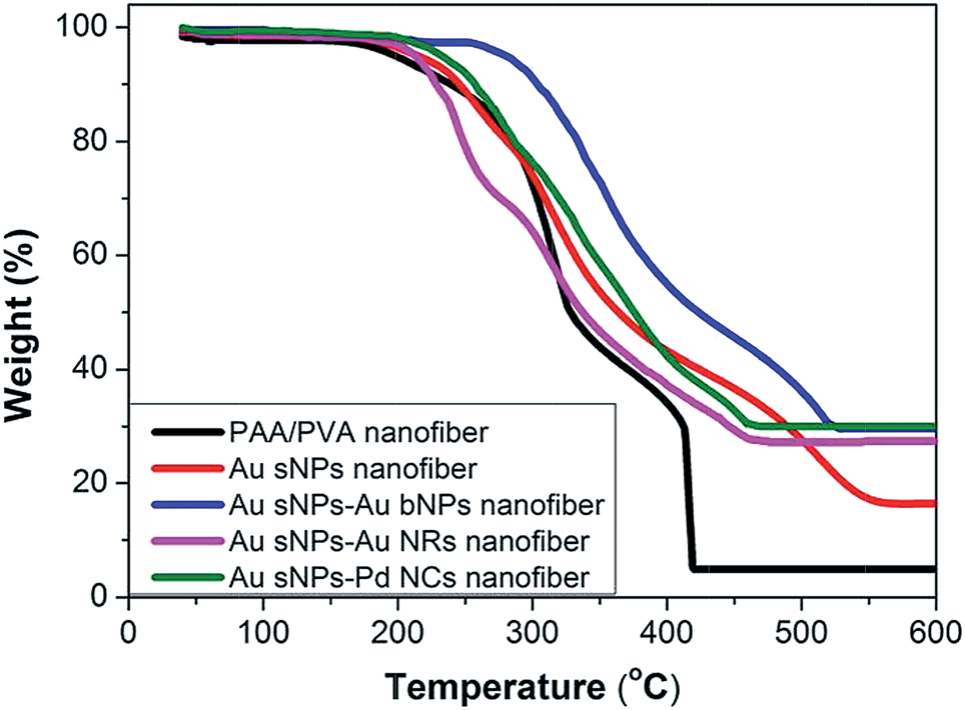
TG curves of the crosslinked PAA/PVA nanofiber, the PAA/PVA nanofibers assembled with Au sNPs, Au sNPs–Au bNPs, Au sNPs–Au NRs and Au sNPs–Pd NCs.

To further confirm the assembly of different NMNPs, XRD as well as TG tests were performed. Compared with the Au sNPs–assembled PAA/PVA nanofibers, the Au sNPs–Au bNPs and Au sNPs–Au NRs nanofibers showed enhanced Bragg diffraction peaks of typical face centered cubic Au crystals (Fig. 3), suggesting more Au nanocrystals were assembled with the electrospun nanofibers after the immersion step.^26^ As for the Au sNPs–Pd NCs nanofibers, new peaks located at 40.1°, 46.5° and 68.0° which refer to the (111), (200) and (220) lattice planes of face centered cubic Pd crystals, were observed because of the presence of Pd NCs.^21^ As shown in Fig. 4, the major weight loss occurred from 200 to 550 °C, which might result from the decomposition of the polymers and the small molecules such as CTAB.^27^ The loading content of the assembled Au sNPs inside the nanofibers is 11.5 wt%, while the loading contents of the assembled Au bNPs, Au NRs and Pd NCs outside the nanofibers are 13.2 wt%, 11.0 wt% and 13.5 wt%, respectively. The weight ratios of the organic component and the metal component are estimated to be 7.7 : 1, 3.0 : 1, 3.4 : 1 and 3.0 : 1 for the PAA/PVA nanofibers assembled with Au sNPs, Au sNPs–Au bNPs, Au sNPs–Au NRs and Au sNPs–Pd NCs, respectively. Moreover, in order to obtain the exact content of the assembled NPs, the metal content on the nanofibers was measured using inductively coupled plasma optical emission spectrometry. As displayed in Table S2,† the loading contents for the Au sNPs, Au bNPs, Au NRs and Pd NCs are 10.5 wt%, 13.0 wt%, 10.5 wt% and 12.8 wt%, respectively, which agree with the TG results.

**Fig. 5:**
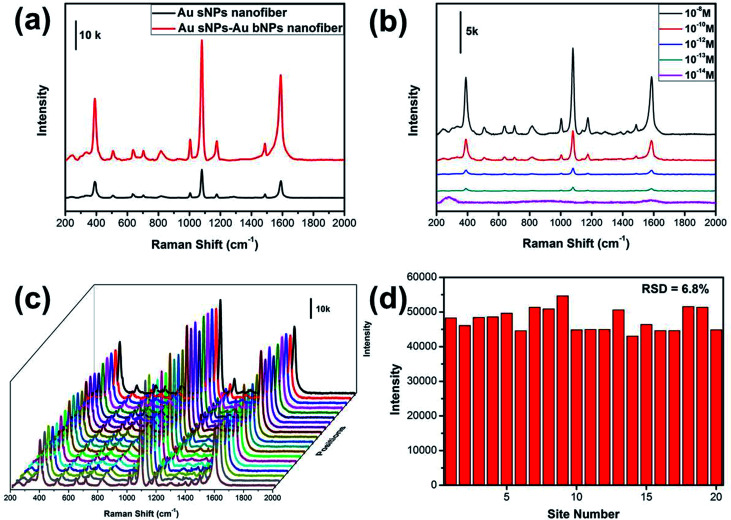
(a) SERS spectra of 0.1 mM 4-ATP molecules collected from the Au sNPs nanofiber and the Au sNPs–Au bNPs nanofiber membrane; (b) SERS spectra of different concentrations of 4-ATP collected from the Au sNPs–Au bNPs nanofiber membrane; (c) SERS spectra of 0.1 mM 4-ATP molecules collected from 20 randomly selected positions of the Au sNPs–Au bNPs nanofiber membrane; (d) the corresponding intensity variation of the 1078 cm^−1^ peaks in (c).

It has been well established that the NMNPs-assembled electrospun nanofiber membrane is excellent 3D SERS substrate, which benefits from the numerous “hot spots” generated from the gaps between neighboring NPs.^28,29^ The SERS performance of the NPs-assembled PAA/PVA electrospun nanofiber was evaluated using 4-ATP as the probing molecule. As shown in Fig. 5a, three obvious peaks at 391, 1078 and 1578 cm^−1^ could be assigned to the a_1_ vibrational modes of 4-ATP, suggesting that the electromagnetic field enhancement dominated the SERS enhancement.^30,31^ Moreover, these characteristic SERS peaks from the Au sNPs–Au bNPs nanofiber membrane are more intense than those from the Au sNPs nanofiber membrane, which may be ascribed to the increased hot spots. Additionally, the size of the Au bNPs may also be responsible for the SERS enhancement.^32,33^ In order to evaluate the actual performance of this 3D SERS substrate (the Au sNPs–Au bNPs nanofiber membrane), the detection of trace amount of 4-ATP was conducted. As shown in Fig. 5b, the SERS intensity decreased with the decreasing concentration of the 4-ATP molecule. When the concentration of 4-ATP reached 10^−13^ M, the featured peaks at 391, 1078 and 1578 cm^−1^ could be easily located. However, there were no distinguishable peaks in the SERS spectrum when the concentration decreased to 10^−14^ M, indicating that the detection limit for the 4-ATP molecule using the Au sNPs–Au bNPs nanofiber membrane was 10^−13^ M, which is superior to many similar materials (Table S1†). The reproducibility of the SERS signals, which is important for the routine analysis, was tested by collecting SERS spectra of 4-ATP from 20 random spots on the Au sNPs–Au bNPs composite nanofibrous membrane (Fig. 5c). There was no obvious difference among these spectra, implying that the reproducible SERS signals are result from the well-distributed NPs. Fig. 5d shows the SERS intensities at 1078 cm^−1^ of the selected spots in Fig. 5c. The relative standard deviation (RSD) was 6.8%, confirming the excellent reproducibility of the SERS substrate.

**Fig. 6:**
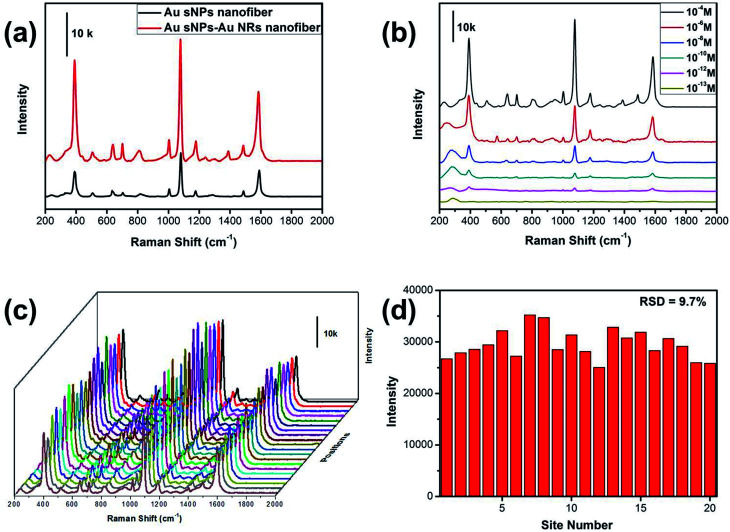
(a) SERS spectra of 0.1 mM 4-ATP molecules collected from the Au sNPs nanofiber and the Au sNPs–Au NRs nanofiber membrane; (b) SERS spectra of different concentrations of 4-ATP collected from the Au sNPs–Au NRs nanofiber membrane; (c) SERS spectra of 0.1 mM 4-ATP molecules collected from 20 randomly selected positions of the Au sNPs–Au NRs nanofiber membrane; (d) the corresponding intensity variation of the 1078 cm^−1^ peaks in (c).

Similarly, the SERS performance of the Au sNPs–Au NRs nanofiber membrane was evaluated as well. As expected, the Au sNPs–Au NRs nanofiber membrane exhibited a better SERS performance than the Au sNPs nanofiber membrane (Fig. 6a). The detection limit for the 4-ATP molecule using the Au sNPs–Au NRs nanofiber membrane was 10^−12^ M, as displayed in Fig. 6b. Because of the uniform distribution of the Au sNPs and the Au NRs assembled both in and on the PAA/PVA nanofibers, this 3D SERS substrate showed a good SERS reproducibility with a RSD value of 9.7% (Fig. 6c and d).

**Fig. 7:**
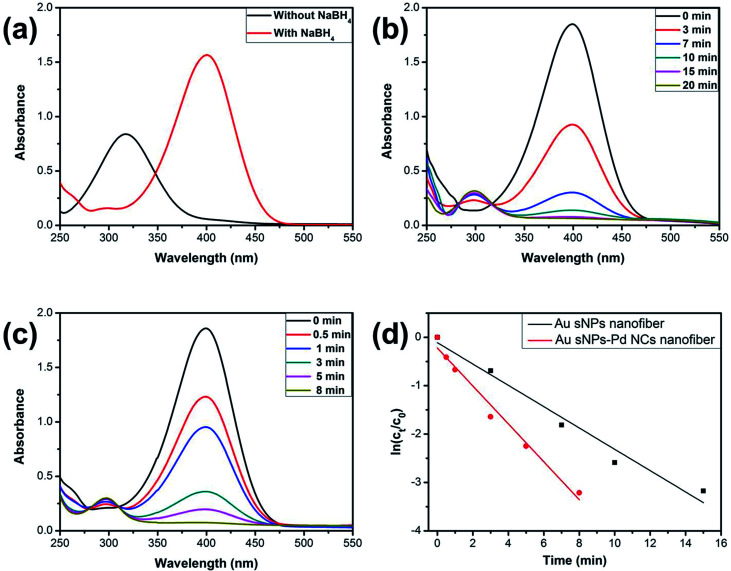
(a) UV-vis spectra of the 4-NP solution before and after the addition of NaBH_4_; (b) UV-vis spectra of the catalytic reduction of 4-NP after adding the Au sNPs nanofiber membrane; (c) UV-vis spectra of the catalytic reduction of 4-NP after adding the Au sNPs–Pd NCs nanofiber membrane; (d) plots of ln(*C*_*t*_/*C*_0_) of 4-NP *versus* time for the reactions using these two kinds of nanocomposite membranes as the catalysts, respectively.

It is known that NMNPs have become promising catalysts for many chemical reactions.^34–36^ Here, the model reduction of 4-nitrophenol (4-NP) to 4-aminophenol (4-AP) was utilized to assess the catalytic property of the NPs-assembled electrospun nanofiber membranes.^37,38^Fig. 7a shows the UV-vis spectra of the 4-NP solution before and after the addition of NaBH_4_. The characteristic absorption peak shifted from 317 nm to 400 nm duo to the formation of 4-nitriphenolate ion after adding NaBH_4_.^37^ However, the reduction cannot further proceed without the presence of catalyst. As shown in Fig. 7b and c, the absorption peaks at 400 nm, which belong to the 4-nitriphenolate ion, gradually became weaker after the addition of the Au sNPs nanofiber or the Au sNPs–Pd NCs nanofiber membrane as the catalysts. New absorption peaks at 300 nm, which belong to the 4-AP, appeared and increased correspondingly. What is more, the reduction process could be finished within 20 min and 8 min for the Au sNPs nanofiber and the Au sNPs–Pd NCs nanofiber membrane, respectively. These results indicate that both the NPs-assembled nanofibrous membranes could efficiently catalyze 4-NP reduction to 4-AP. Since there was excess NaBH_4_ in the solution, the reaction could be assumed to follow pseudo-first-order kinetics. Therefore the apparent rate constant (*k*_app_) could be calculated using the equation ln(*C*_*t*_/*C*_0_) = −*k*_app_*t*, where the *C*_*t*_ and *C*_0_ are the apparent and initial concentrations of 4-NP, and *t* represents the reaction time. The calculated values of *k*_app_ were 3.7 × 10^−3^ s^−1^ and 6.5 × 10^−3^ s^−1^ for the Au sNPs nanofiber and the Au sNPs–Pd NCs nanofiber, respectively. This means that the Au sNPs–Pd NCs nanofibrous membrane exhibited better catalytic performance, which could be ascribed to the higher content of the assembled catalytic NMNPs.

## Conclusion

In this work, various NMNPs with different sizes, different elemental compositions and different shapes were successfully assembled both into and onto the PAA/PVA electrospun nanofiber by simple mixing and immersion steps. The NPs were found uniformly distributed in or on the nanofibers. Owing to the increased loading content, the obtained composite nanofibrous membrane assembled with two different kinds of NPs exhibited improved SERS and catalytic performance. Furthermore, this new design strategy and fabrication protocol of the hybrid NPs-assembled nanofiber membrane could be readily extended to the engineering of other functional nanofibrous membrane.

## Conflicts of interest

There are no conflicts to declare.

## Supplementary Material

RA-008-C8RA00665B-s001
