# Supplementary material for: Electrospun nanofiber templated assembly of hybrid nanoparticles
Source: RSC Adv. 2018 Mar 5;8(17):9344–52. doi: 10.1039/c8ra00665b (PMC9078683; doi:10.1039/c8ra00665b)
Supplement: RA-008-C8RA00665B-s001 [file RA-008-C8RA00665B-s001.pdf]

Electronic Supplementary Material (ESI) for RSC Advances.
This journal is © The Royal Society of Chemistry 2018

Supporting Information

Electrospun nanofiber templated assembly of hybrid nanoparticles

Zhicheng Liu[a,c], Zhaodong Yan[a], Lu Bai[b]*

a School of Materials Science and Engineering, North University of China, Taiyuan 030051, China

b School of Chemical Engineering and Technology, North University of China, Taiyuan 030051, China

c Department of Mechanical Engineering, National University of Singapore, Singapore, 117574, Singapore

* Corresponding author: bailu0919@gmail.com

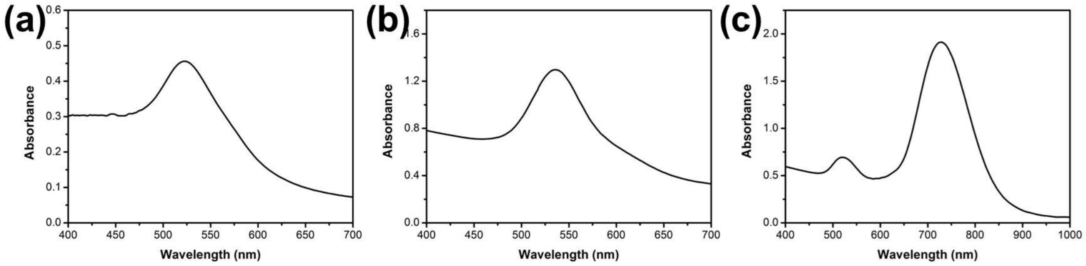

Fig. S1 UV-vis spectra of (a) Au sNPs, (b) Au bNPs, (c) Au NRs.

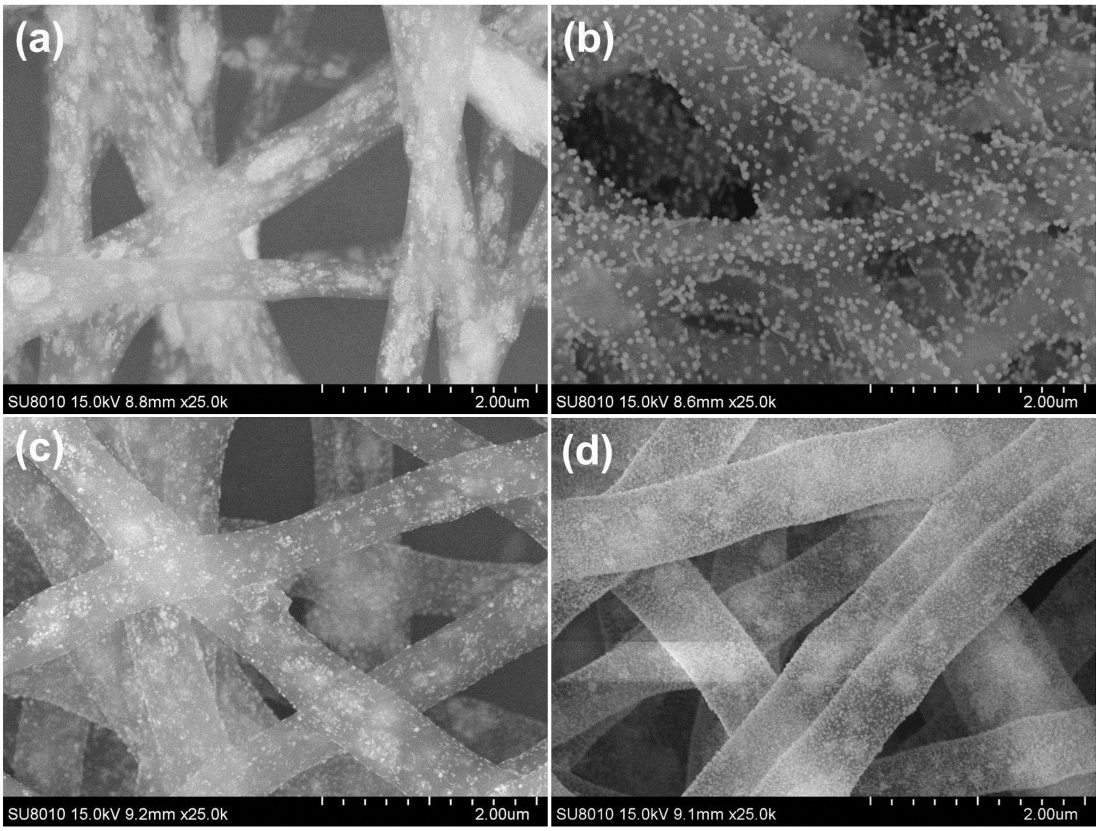

Fig. S2 Low magnification SEM images of the PAA/PVA electrospun nanofibers assembled with (a) Au sNPs, (b) Au sNPs-Au bNPs, (c) Au sNPs-Au NRs and (d) Au sNPs–Pd NCs.

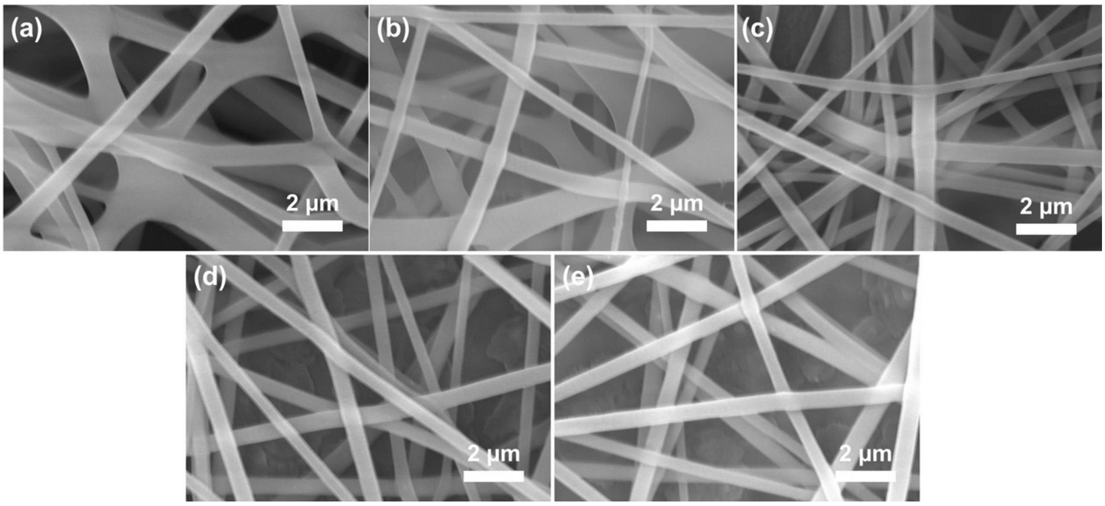

Fig. S3 SEM (S-4300, nanofibers were sputtered with a gold layer) images of the PAA/PVA electrospun nanofibers assembled with Au sNPs. The needle-to-collector distances are (a) 10 cm, (b) 15 cm, (c) 20 cm, (d) 25 cm and (e) 30 cm, respectively.

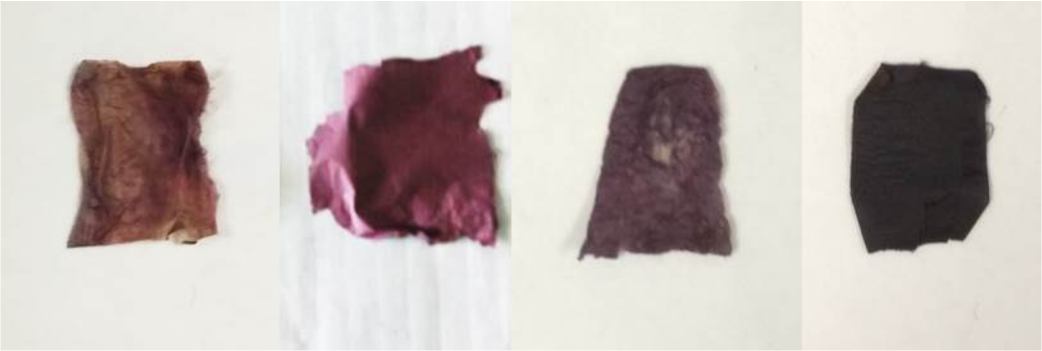

Fig. S4 Photographs of the PAA/PVA electrospun nanofibers assembled with different NPs: from left to right, Au sNPs, Au sNPs-Au bNPs, Au sNPs-Au NRs and Au sNPs–Pd NCs. The size of the membranes is about 1.5 cm × 1.5 cm.

Table S1 Comparison of the Au sNPs-Au bNPs composite nanofibrous membrane for 4-ATP detection with other nanofiber membranes.

Refs.	Materials	Detection limit
1	Ag NPs assembled on PEI/PVA nanofibers	10^{-8} M
2	plate-like Ag NCs gown on nanofibers	10^{-10} M
3	Ag NPs assembled on PE nanotubes	10^{-8} M

4	Ag NP functionalized glass fiber	5×10^{-8} M
5	Ag NPs decorated on 3D TiO_2 film	10^{-7} M
6	Ag NCs assembled tapered fiber probe	10^{-7} M
7	Au-coated magnetic nanoparticles	10^{-9} M
8	Au NPs decorated inverse opal capillary	10^{-9} M
9	Au NPs decorated on PAA/PVA nanofibers	10^{-8} M
10	ZnO nanofibers deposited on silver foil	10^{-12} M
11	Au NPs decorated on cellulose nanofibers	10^{-12} M
This work	Au sNPs-Au bNPs composite nanofibers	10^{-13} M

References for Table S1:

[1] T. Yang, J. Ma, S. J. Zhen and C. Z. Huang, *ACS Appl. Mater. Interfaces.*, **2016**, 8, 14802-14811.

[2] P. Jia, J. Qu, B. Cao, Y. X. Liu, C. Luo, J. H. An and K. Pan, *Analyst*, **2015**, 140, 5190-5197.

[3] L. B. Huang, Y. Zhou, S. T. Han, Y. Yan, L. Zhou, W. Chen, P. Zhou, X. F. Chen and V. A. L. Roy, *Small*, **2014**, 10, 4645-4650.

[4] M. Kurita, R. Arakawa and H. Kawasaki, *Analyst*, **2016**, 141, 5835-5841.

[5] H. C. Dai, Y. J. Sun, P. J. Ni, W. D. Lu, S. Jiang, Y. L. Wang, Z. Li and Z. Li, *Sens. Actuators, B*, **2017**, 242, 260-268.

[6] Z. L. Huang, X. Lei, Y. Liu, Z. W. Wang, X. J. Wang, Z. M. Wang, Q. H. Mao and G. W. Meng, *ACS Appl. Mater. Interfaces.*, **2015**, 7, 17247-17254.

[7] J. F. Wang, X. Z. Wu, C. W. Wang, Z. Rong, H. M. Ding, H. Li, S. H. Li, N. S. Shao, P. T. Dong, R. Xiao and S. Q. Wang, *ACS Appl. Mater. Interfaces.*, **2016**, 8, 19958-19967.

[8] X. W. Zhao, J. Y. Xue, Z. D. Mu, Y. Huang, M. Lu and Z. Z. Gu, *Biosens. Bioelectron.*, **2015**, 72, 268-274.

[9] Z. C. Liu, Z. D. Yan, L. Jia, P. Song, L. Y. Mei, L. Bai and Y. Q. Liu, *Appl. Surf. Sci.*, **2017**, 403, 29-34.

[10] W. Song, W. Ji, S. Vantasin, I. Tanabe, B. Zhao and Y. Ozaki, *J. Mater. Chem. A*, **2015**, 3, 13556-13562.

[11] R. Chen, L. Zhang, X. Li, L. Ong, Y. G. Soe, N. Sinsua, S. L. Gras, R. F. Tabor, X. Wang and W. Shen, *ACS Sens.*, **2017**, 2, 1060-1067.

Table S2 ICP results of the the PAA/PVA electrospun nanofibers assembled with different NPs.

Sample	Au	Pd	Loading Contents
Au sNPs (10.18 mg)	1.07 mg	N/A	Au sNPs: 10.5 wt%
Au sNPs-Au bNPs (9.80 mg)	2.30 mg	N/A	Au bNPs: 13.0 wt%
Au sNPs-Au NRs (10.62 mg)	2.23 mg	N/A	Au NRs: 10.5 wt%
Au sNPs–Pd NCs (10.25 mg)	1.06 mg	1.31 mg	Pd NCs: 12.8 wt%
